# Peroral direct cholangioscopy and snare technique for stray bile duct stone for patients who have undergone bile duct jejunostomy

**DOI:** 10.1055/a-2601-0038

**Published:** 2025-06-26

**Authors:** Nobuhiko Fukuba, Hiroyuki Fukuhara, Yoshiko Takahashi, Yasuhide Kodama, Masaki Onoe, Shuichi Sato, Shunji Ishihara

**Affiliations:** 1 175764 Department of Gastroenterology and Hepatology, Shimane University Faculty of Medicine Graduate School of Medicine, Izumo, Japan; 2 73826 Internal Medicine, Izumo City General Medical Center, Izumo, Japan; 3 73826 General Medicine, Izumo City General Medical Center, Izumo, Japan

A male patient over 80 years old, who underwent a pancreaticoduodenectomy 7 years prior, was presented with transient abdominal pain. Computed tomography (CT) findings indicated the presence of a common bile duct stone, thus a balloon-assisted enteroscopy-assisted endoscopic retrograde cholangiopancreatography (BAE-ERCP) procedure was performed. However, the stone could not be identified in the cholangiography images due to gas reflux, while sweeping with a basket was also ineffective.

After 2 months, the patient experienced abdominal pain recurrence and subsequent CT scanning revealed the previously identified stone. It was determined that BAE-ERCP alone would not be able to identify the stone, thus a peroral direct cholangioscopy (PDCS) examination was performed. The procedure was initiated with the insertion of a double-balloon endoscope (EI-580BT, Fujifilm, Tokyo) into the area of the previous bile duct jejunal anastomosis, followed by its removal with the overtube remaining ( Video 1 ). Next, balloon dilation was performed, and a slit was made in the overtube, through which a small-caliber endoscope (SCE) (EG-740N, Fujifilm, Tokyo) was inserted. The SCE was then fitted tightly into the anastomosis and observations of the interior of the bile duct revealed that the stone had become trapped inside during the suction procedure, similar to a ball-check valve ( Fig. 1 ). The stone was grasped using a snare (Snare Master, Olympus), then crushed and removed by aspiration with the SCE ( Fig. 2 ).

**Fig. 1:**
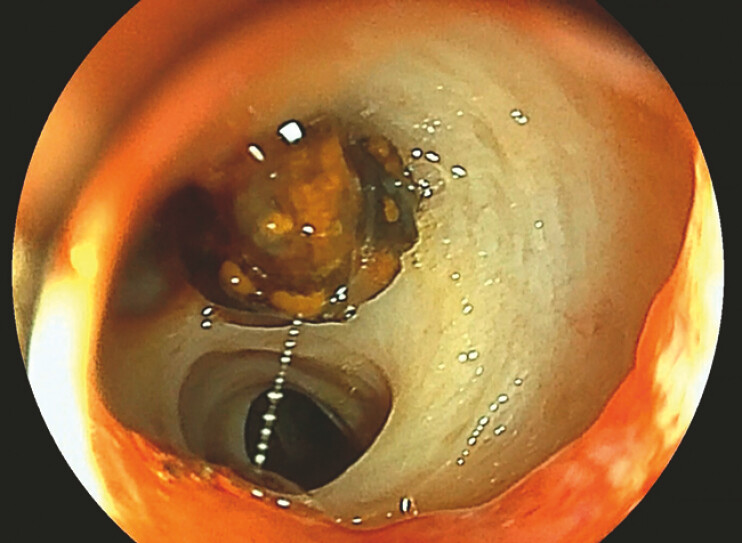
The stone had become trapped within the anastomosis, similar to a ball-check valve.

**Fig. 2:**
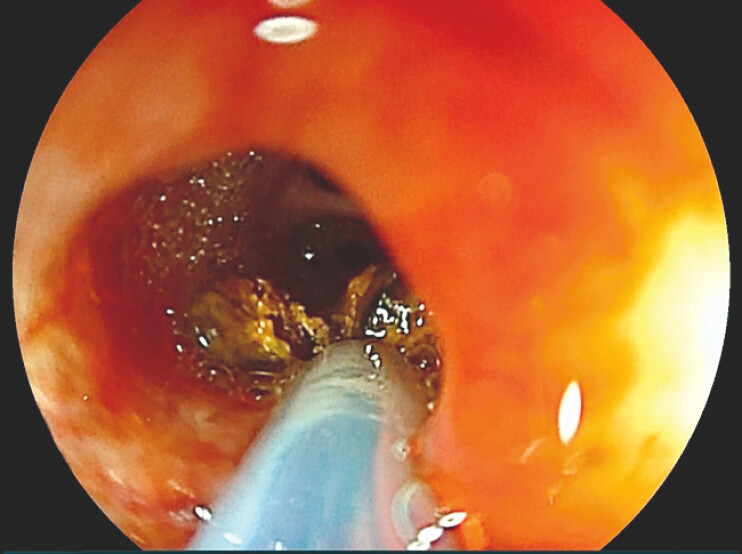
The stone was easily grasped with the snare and split into two pieces with little force required.

During the anastomosis procedure, observation of the internal portion of the bile duct with the SCE revealed a trapped stone, similar to a ball-check valve. A snare (Snare, Fujifilm) was used to grasp the stone, allowing it to be crushed and then removed by aspiration with the SCE.Video 1

The effectiveness of BAE-ERCP for cases with a reconstructed intestine has been reported, though stone removal is generally difficult 1 2 3 4 . The SCE used has a 2.4-mm channel diameter, which facilitated the utilization of a snare and allowed the stone pieces to be removed by suction. It is difficult to identify floating stones in bile duct jejunal anastomosis cases using cholangiography, due to gas reflux. PDCS was found to be effective in the present case in identifying and efficiently removing the stone.

Endoscopy_UCTN_Code_TTT_1AP_2AD

## Notes

Endoscopy E-Videos https://eref.thieme.de/e-videos
*E-Videos* is an open access online section of the journal *Endoscopy* , reporting on interesting cases and new techniques in gastroenterological endoscopy. All papers include a high-quality video and are published with a Creative Commons CC-BY license. Endoscopy E-Videos qualify for HINARI discounts and waivers and eligibility is automatically checked during the submission process. We grant 100% waivers to articles whose corresponding authors are based in Group A countries and 50% waivers to those who are based in Group B countries as classified by Research4Life (see: https://www.research4life.org/access/eligibility/ ). This section has its own submission website at https://mc.manuscriptcentral.com/e-videos .
